# Comparative evaluation of pain perception with a new needle-free system and dental needle method in children: a randomized clinical trial

**DOI:** 10.1186/s12871-021-01524-1

**Published:** 2021-12-01

**Authors:** Halenur Altan, Melek Belevcikli, Alem Coşgun, Osman Demir

**Affiliations:** 1 Faculty of Dentistry, Department of Pediatric Dentistry, Tokat Gaziosmanpaşa University, 60250 Tokat, Turkey; 2 Faculty of Dentistry, Department of Pediatric Dentistry, Zonguldak Bulent Ecevit University, Zonguldak, Turkey; 3 Faculty of Medicine, Department of Biostatistics, Tokat Gaziosmanpaşa University, Tokat, Turkey

**Keywords:** Children, Dental needle, Needle-free injection system, Pain, Pulpotomy

## Abstract

**Background:**

Pain control during dental procedures is one of the most important topics related to behavior management in children. This study aims to compare the pain perception associated with a needle-free system (Comfort-In™) and the dental needle method during filling and pulpotomy treatments in children.

**Methods:**

The study included teeth that required treatment (pulpotomy or filling treatment) in 56 patients aged 4 to 11 years with no systemic problems or history of allergy. Patients were randomly divided into the needle-free system group (filling treatment, *n* = 13; pulpotomy, *n* = 15) and dental needle method group (filling treatment, *n* = 14; pulpotomy, n = 14). For pulpotomy and filling treatment performed with 0.3 mL anesthesia, the active ingredient of which is 2% lidocaine and 1/80000 epinephrine. The patients’ behavior before the procedure was evaluated by a pediatric dentist using the Frankl Behavior Scale. The pain intensity was assessed Immediately after injection (induction), during treatment (treatment), and at the end of the treatment (post treatment) by the Wong-Baker Faces Pain Scale.

**Results:**

The median (IQR-InterQuartile Range) induction pain value was 6[3-8] and 2[0-4] in dental needle method and needle-free system respectively, *p* < 0.001). In filling and pulpotomy treatment group, no difference between the needle and needle-free group for treatment and post-treatment pain values.

**Conclusions:**

For pulpotomy and filling treatment, needle-free system performed with 0.3 mL anesthesia was found as effective as infiltrative anesthesia with a dental needle method.

**Trial registration:**

ClinicalTrials.gov, NCT04653974. Registered 4 December 2020 – Retrospectively registered.

## Background

Needle-related pain (NRP) occurs most frequently during anesthesia in dentistry. In children, the pain from NRPs can produce an emotional and negative cognitive response toward the procedure [1]. Parents and children may also delay treatment or avoid dental treatment, exhibit weak cooperation, or develop needle phobia [2, 3]. In addition, patients often experience more fear at the sight of a needle during the administration of local anesthetic than from the treatment itself [4]. Therefore, dentists have attempted to minimize the intensity of NRP associated with any dental problem [5].

Pressure injector systems can be applied as an alternative to conventional dental needle method [6, 7]. Jet-injector systems are needle-free systems that work with the principle of applying anesthetic solution with pressure to penetrate the tissues with spring-loaded devices, in a manner reinforced with pressurized air or gas [8, 9].

To address the patients’ injection fear, jet-injectors are designed to eliminate the pain and fear associated with conventional dental injectors and syringes [6]. In this study, we aimed to compare the pain perception in filling and pulpotomy treatments with a new needle-free system (Comfort-In™^)^ and with dental needle method in a pediatric population.

## Methods

The study protocol was approved by the Tokat Gaziosmanpaşa University Clinical Research Local Ethics Committee (18-KAEK-089), registered at ClinicalTrials.gov (NCT04682080). The data were presented in accordance with the CONSORT statement. Before the study, written informed consent was obtained from the each parent of the children included in the study stating that they accepted the treatment. This study was performed in accordance with the ethical standards of the Declaration of Helsinki (1964) and its subsequent amendments.

### Participants selection

This study was performed among children aged 4-11 years who required filling or pulpotomy treatments in their primary molars. Children following the dentist’s directions during the clinical examination and who had a periapical film taken on radiographic examination without crying were accepted “positive” and “definitely positive” dental behavior according to the Frankl Behavior Scale (FBS). In addition, the dental behavior of the children was checked before the treatment 1 week later after the dental examination was performed. All dental equipment and operations were introduced using the “tell-show-do” technique. Then, the injection was described to the patients using appropriate words (statements such as “put the tooth to sleep”). (Table 1).

**Table 1:** Treatment indications and contraindications

Treatment options		Restorative treatments (pulpotomy and filling treatment) contraindications
Pulpotomy treatments indications	Filling treatments indications	
• Tooth with deep caries without pulp exposure • Carious or traumatic pulp exposure with transitory thermal and /or chemical stimulated pain • Physiologic mobility, healthy soft tissue, and no percussion sensitivity • İntact continuous ligament space and intact periapical and/or furcation bone	• Tooth with caries without pulp exposure • No spontaneous pain and/or lost the pain when the stimulus disappears. • Physiologic mobility, healthy soft tissue, and no percussion sensitivity • Intact continuous ligament space and intact periapical and/or furcation bone	• Tooth with irreversible pulpitis, determined as continuous bleeding exceeding 5 minutes, dark to purple blood color or pulp necrosis • Radiographic periapical or interradicular radiolucencies • Tooth with internal resorption/external resorption • If the patient’s age is exfoliating the tooth • When root resorption is 2/3 of root length. • If tooth infection affects general health • If tooth infection affects permanent tooth • If the bone thickness on the permanent tooth is less than 1 mm. • If there is a loss of matter in teeth that cannot be restored

Children who needed dental treatment were randomly divided into two groups (filling and pulpotomy). The same operator (MB) was administered all dental injections, a pediatric dentist with 2 years of experience in using the needle-free system (Comfort-In™).

Supplemental anesthetics due to pulpal inflammation: When the coronal pulp is amputated, the remaining radicular tissue must be considered vital without radiographic signs of infection or pathologic resorption. If the child feels pain while the coronal pulp is amputated, additional anesthesia is administered. Excessive hemorrhage-related pulpal inflammation that cannot be controlled in 5 min after anesthesia is provided, these patients will be excluded.

### Inclusion and exclusion criteria

Inclusion Criteria: Aged between 4 and 11 years, having no developmental or systemic disorder or no history of allergy, having “positive” or“ definitely positive”; cooperation level according to the Frankl Behavior Scale (FBS), having sufficient mouth opening, operation only on primary teeth, having decayed teeth that require anesthesia.

Exclusion criteria: “Negative” or “definitely negative” behavior rating according to the Frankl Behavior Scale (FBS), operating only on permanent teeth.

Supplemental anesthetics due to pulpal inflammation: When the coronal pulp is amputated, the remaining radicular tissue must be considered vital without radiographic signs of infection or pathologic resorption. If the child feels pain during amputation of coronal pulp, additional anesthesia is administered. Patients with excessive hemorrhage-related pulpal inflammation that cannot be controlled in 5 min after anesthesia will be excluded.

### Anesthesia protocols

Prepared tissue at the injection site was cleaned with sterile dry gauze and a small quantity of topical anesthetic (Lidocaine 10%, Vemcain, Turkey) and kept in place for at least 1 min.

### Needle-free system

**Fig. 1:**
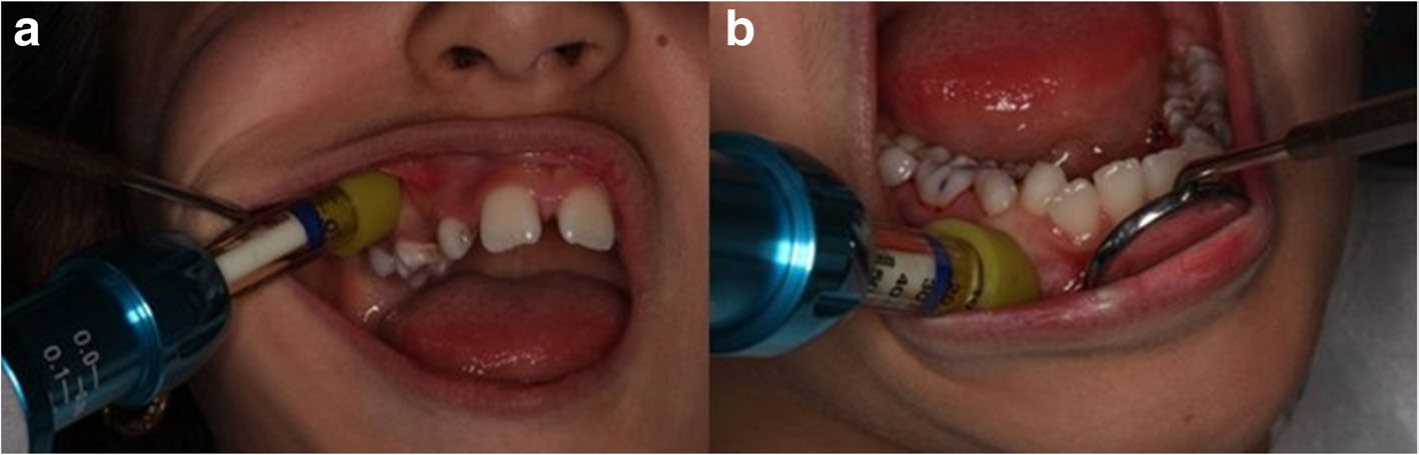
**a** Needle-free system (Comfort-In™) on maxilla. **b** Needle-free system (Comfort-In™) on mandible

The protocol for the needle-free system (Comfort-In™, Mika medical, Korea) group was as follows. (Fig. 1a-b).

The prepared injector was placed firmly on the skin/mucosa. The injector was adjusted while avoiding increasing the distance between the bone and injector because this may cause injury to the bone since the anesthetic exits the injector at high speed. The equipment had a pressurized spring and a silicone cap (recto cap) coupled with an ampoule containing the anesthetic solution for preserving the periodontal tissues. The injector was positioned 90° to the maxilla or mandible with slight compression next to the gingival band inserted at the target tooth. Anesthesia was administered by pressing a button to release the anesthetic solution. The children and parents were informed about the “pop” noise produced by the equipment during release of the anesthetic solution to prevent fear. In needle-free system, 2% lidocaine with 1/80.000 epinephrine (Lidocaine, Colombia) was injected using the Comfort-In™ system. Approximately 0.2 mL of the anesthetic solution was deposited for filling and 0.3 mL for pulpotomy treatment with a pressure of 2000 psi to the cutaneous/subcutaneous tissue in less than 2 s. After injection, the injector tip was kept pressed against the injection site for a few seconds.

### Dental needle method

In dental needle method, 2% lidocaine with 1/80.000 epinephrine (Lidocaine, Colombia) was injected using a 27G, 40-mm, disposable syringe with a needle (Genject, Turkey).

The needle was oriented such that the bevel faced the bone. The lip was lifted, and mucosa was stretched. The syringe was held parallel to long axis of tooth, and the needle penetrated mucobuccal fold and was inserted to the depth of the apices of the buccal roots of teeth. The depth of penetration was only a few millimeters. Approximately 0.2 mL of the anesthetic solution was deposited for filling and 0.3 mL for pulpotomy treatment. The injection rate was standardized to ~ 1 mL/min for the control group. Care was taken to avoid ballooning of tissues. The dental procedures were initiated after 5 min.

During treatment, the cotton roll technique was used to isolate the oral cavity.

### Pain assessment

The facial expressions rating (Wong-Baker) scale consists of 6 facial expressions rated from 0 to 10 according to pain intensity. It is a valid and reliable scale in assessing acute pain in school-age children since children with a bit of explanation efficiently and quickly understand it. In the evaluation of the scores obtained from the scale, while the values between 0 and 4 indicate mild pain, the values between 4 and 6 indicate moderate pain, the values between 6 and 8 indicate severe pain, and the values between 8 and 10 indicate unbearable pain [10].

In both groups, the children were asked to rate their pain intensity by choosing the closest statement on the Wong-Baker Pain Scale at three-time points: immediately after injection (induction), during treatment (treatment), and at the end of the treatment (post-treatment).

#### Induction

Immediately after administering anesthesia, the patients were told to choose the color and facial expression closest to them by considering how they felt while their teeth were putting to sleep and whether they perceived pain. The selected pain intensity represented “Induction”.

#### Treatment

After the anesthesia was administered, it waited for 5 min, and the necessary dental procedure was started. In the cavity preparation in filling treatment, pulp extirpation in pulpotomy treatment, the children were asked to choose the facial expression closest to them by considering whether their teeth were hurt. The intensity of pain children chose represented “Treatment”.

#### Post-treatment

At the end of the dental treatment, they were asked whether that tooth was hurt and asked to choose a facial expression closest to them by again from the same scale. The intensity of pain children chose represented “Post-treatment”.

### Power analysis

The study design was a randomized controlled and cross-over clinical study. With an α = 0.05, 1-β = 0.8 and a Cohens’s d = 0.63 [11], the minimum sample size was estimated at 64 per group. The sample power calculation was performed using t test family in G*Power 3.1.9.6 program (Universität Kiel, D) [12].

### Statistical analysis

Quantitative variables are presented as average and standard deviation values, while qualitative variables are presented as percentages(n) or as median (1th quartile,3th quartile). Percentage analysis was performed for the distribution of tooth and the choice of the anesthetic technique. The effect of the type of anesthesia and of its interaction with the treatment type was tested by multiple ANOVA. Mann-Whitney U test was used to compare the pain scores according to treatment and anesthesia types. *p* values were considered statistically significant when they were below 0.05. Calculations were performed using available statistical software (IBM SPSS Statistic 19; SPSS Inc., an IBM Co., Somers, NY).

## Results

**Fig. 2:**
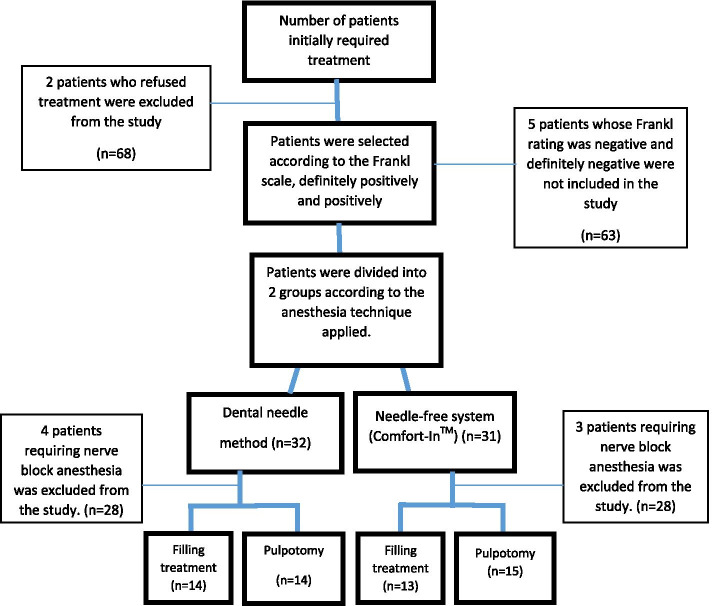
Study flow chart

A total of 70 patients were evaluated following the exclusion criteria, and 56 children (25 boys and 31 girls) aged 4–11 years (needle-free system 6.36 ± 1.06 and dental needle method 7 ± 2.10) were included in this study (Table 2). For needle-free system, 16 girls and 12 boys, 15 girls and 13 boys were included in this study for dental needle method (Fig. 2). No difference between tooth numbers was detected in needle-free system and dental needle method (Table 2).

**Table 2:** Tooth number distributions according to the treatments and anesthesia types

Tooth Number	Filling		P^a^	Pulpotomy		P^a^	Whole Sample		P^a^
	Needle-free system	Dental Needle Method		Needle-free system	Dental Needle Method		Needle-free system	Dental Needle Method	
54-64^b^	4(26,7)	2(14,3)	0.550	4(26,7)	4(28,6)	0.589	8(26,7)	6(21,4)	0.336
55-65^c^	5(33,3)	7(50)		4(26,7)	4(28,6)		9(30)	11(39,3)	
74-84^d^	3(20)	4(28,6)		2(13,3)	4(28,6)		5(16,7)	8(28,6)	
75-85^e^	3(20)	1(7,1)		5(33,3)	2(14,3)		8(26,7)	3(10,7)	

^a^Pearson Chi Square, n(%); ^b^Right primary maxillary first molar-Left primary maxillary first molar; ^c^Right primary maxillary second molar-Left primary maxillary second molar; ^d^Left primary mandibular first molar-Right primary mandibular first molar; ^e^Left primary mandibular second molar-Right mandibular primary second molar

### Pain assessment

There was a significant difference between the needle-free system and dental needle method during administered anesthesia (*p* < 0.001). The median (IQR) induction pain value was 6[3-8] and 2[0-4] in dental needle method and needle-free system respectively, p < 0.001) (Table 3). In filling treatment group, no difference between the needle and needle-free group for treatment and post-treatment pain values. Similarly, in pulpotomy treatment group, no difference between the needle and needle-free group for treatment and post-treatment pain values (Table 3). When adjusting the treatment effect on the anesthesia group, the model was not found predictive (*p* = 0.648).

**Table 3:** Distribution of pain scores according to the treatments and anesthesia types

Treatment	Filling treatment			Pulpotomy			Whole Sample		
Anesthesia	Needle-free System	Dental Needle Method	p	Needle-free System	Dental Needle Method	p	Needle-free System	Dental Needle Method	p
Induction	2[0-4]	6[6-8]	**0.004**	2[0-4]	6[2-10]	**0.037**	2[0-4]	6[3-8]	**< 0.001**
Treatment	2[0-4]	0[0-4]	0.270	4[0-6]	2[2-4]	0.780	2[0-4]	2[0-4]	0.341
Post-treatment	0[0-2]	0[0-2]	0.813	0[0-2]	2[0-2]	0.533	0[0-2]	0[0-2]	0.481

p: Between-group comparisons for anesthesia group (Mann Whitney U test)

## Discussion

In the present study, Comfort-In™ needle-free injection system was as effective as gold standard dental needle method for filling and pulpotomy treatments. In addition, less pain was found during anesthesia administered with Comfort-In™ needle-free injection system than dental needle method. To the best of our knowledge, this study is the first to compare the pain perception of dental needle method and needle-free injection system (Comfort-In™) during all treatment at three different times in children.

The child’s response to dental treatment is complex, and many factors affect this process. The child’s age, temperament, level of anxiety, parental anxiety, previous dental experience are among the factors that influence the child’s response to the treatment [13, 14]. The pain perception is affected by the physical factors and the psychological and mental factors of the child [15]. In very young children, other stimuli such as appearance of dental equipments, smell and taste of drugs-sprays, vibration, pressure, difficulty breathing, and limited mouth opening, etc., can also cause significant discomfort treatment experience [16]. Dental injection with needle is one of the major factors which trigger dental anxiety and fear during dental anxiety [6]. In our study, lower pain scores during anesthesia at the needle-free system group than dental needle method group indicate that needle negatively triggers pain perception.

In the literature comparing various types of needle-free system and dental needle method, there was no consensus for which injection type was less painful during anesthesia. Makade et al. [17] reported that the pain perception during anesthesia was higher in dental injector method than jet injection system (Madajet) in adults. In a study comparing pain during dental-injection method and jet injection system (Injex) in eighty-seven coopere children, it was reported that a higher level of pain occurred in jet injection system [18]. A study involving one hundred children aged between 3 and 12 years reported that pain perception was significantly reduced with the Madajet XL needle-free system. Ocak et al. [4] indicated that the jet injection system (Injex) caused less pain during injection compared to dental injector method. Oliveira et al. [19] found no difference in pain perception during anesthesia with the needle-free injection (Comfort-In™) system and dental injection in adults. In this study, needle-free system and dental needle method presented similar results in pain perception during treatment and end of the treatment in both filling and pulpotomy.

Needle-free systems are used successfully in curettage and scaling, gingivectomy, biopsy and abscess drainage, pre-anesthesia and filling [9, 11, 20]. There are different opinions about the success of jet injection systems in providing pulpal anesthesia in the literature. Soft tissue anesthesia was considered “good”; however, the success rate of pulpal anesthesia for the lateral maxillary teeth was found to be weak in 13% of the patients with the Syrijet system [4]. An additional injection was required to reach the sufficient anesthesia level in 80.5% of children with the Injex system [18]. Makade et al. [17] stated that restorative procedures such as those involving Class I and Class II filling and vital pulp treatments could be efficiently completed with the anesthesia provided by needle-free systems. In this study, the measurement of similar pain values in filling treatment and pulpotomy treatment showed that adequate anesthesia depth was achieved with the Comfort-In™ injection system.

According to the present results, for pulpotomy treatment performed with 0.3 mL anesthesia, the active ingredient of 2% lidocaine and 1/80000 epinephrine was found effective as dental needle method. Theocharidou et al. [21] with 0.3 ml articaine 4% and Oliveira et al. [19] 1 mL lidocaine 2% reported that efficiency of anesthesia was significantly reduced 15 mins after anesthesia administered. The onset time of anesthetic effect in Comfort-In™ injection system was shorter than dental needle method, and total anesthesia duration was higher in infiltrative anesthesia with dental needle method. The remarkable increase in pain scores during pulpotomy in needle-free system may be associated with the rapid decrease in the effectiveness of the anesthesia.

Dental treatment under rubber dam isolation is ideal, but it is not used to provide standardization as it may cause application difficulties in young children. Anesthesia is required to numb the gingiva in palatinal / lingual areas in rubber dam application. One of the limitations of this study was that the rubber dam was not used to prevent bias as it affects children’s pain perception and cooperation levels. Therefore, in future studies, pain intensity during rubber-dam application can be compared with other times. The second limitation was that the pain reported after anesthesia might be affected by the first applied anesthesia method. Third, a split-mouth design may affect the behavior and perception of pain during dental anesthesia, especially when highly anxious young patients may be affected more in early treatment sessions. In this research, a parallel design was implemented considering this point. Fourth limitation of this study are being open label study with no pre-hoc defined primary outcome and having small sample size with heterogeneity (two types of treatment).

Also, The effect of the residual fear of dental care and individual oral health status on needle –free injection system need to research to increase the level of evidence for the Comfort-In™ system.

## Conclusions

One of the most important factors that cause fear during dental treatment in children is fear of injection. The child’s experience of painless and fearless dental treatment will make this person more comfortable during dental procedures in the future. For pulpotomy and filling treatment performed with 0.3 mL anesthesia, the active ingredient of 2% lidocaine and 1/80000 epinephrine was found as effective as infiltrative anesthesia with a dental needle method. Comfort-In™ needle-free system reduced the injection pain during anesthesia administered. The dental needle method is more effective than the needle-free system in pulpotomy.

## Data Availability

The datasets used and/or analyzed during the current study are available from the corresponding author on reasonable request.

## Abbreviations

NRP: Needle-related pain
FBS: Frankl Behavior Scale
IQR: InterQuartile Range
